# Brawn or Brains—Or Both?

**Published:** 1921-01-15

**Authors:** 


THE PUBLIC WELL-BEING—(*continued*).

### Brawn or Brains—or Both?

### HOW TO EDUCATE THE WORKING-CLASS BOY.

THE importance of the physical side of education has been urged with so much emphasis in these columns that we have read with interest the rather daring address on the subject delivered a few days ago by Mr. M. F. Cahill to the Educational Conference in London. With much that he said we are in hearty agreement, though we cannot go all the way with him in his sweeping dictum that "all that a working boy now needs is a broad back, a healthy body, and a habit of obedience."

That remark comes very much like an echo from the past. It is a waste of time in these days to put such a proposition before the British working classes, and Mr. Cahill would no doubt have received short shrift if he had ventured to do so before a working-class audience. But, besides not being practical politics, it is a fundamentally unsound proposal. The habit of obedience is useful enough for all boys, whether they are the sons of working men or of millionaries; but to suggest that blind subservience to his master and his fate is the only factor that counts in the intelligence of a boy who will probably become a manual worker is to bring us back to the dark ages, and to insult the legitimate hopes of mankind. To take this consideration alone, the use of machinery is a main factor in the improvement of working conditions and the general prosperity of the race. This is demonstrated by Dr. Marshall in his "Principles of Economics," where he shows how the tendency of industrial progress has been to relieve mankind of its more degrading and purely mechanical tasks, relegating them to the unemotional care of a machine. The result has

**THE PUBLIC WELL-BEING—(*continued*).**

been to set men's minds free for worthier objects; and this is undoubtedly seen in the increase of mental as opposed to physical interests among workmen.

We cannot believe that Mr. Cahill really intended his dictum to be carried to its logical conclusion to reduce the worker to the condition of a stupid serf, for the practical result, excluding the ethics of the matter, would be to lower the standard of life for the whole community by reducing the skill and capacity of the largest section. After all, the habit of obedience hardly enables a man by some magic formula to become a practical electrician. The acquiring of knowledge, if modern civilisation is to be maintained, is essential, as well as a developing impulse that it is too late now to attempt to destroy. We suppose that what Mr. Cahill was really striving to drive home by the familiar method of hyperbole and epigram was the danger of laying too much stress in present-day education on the mental side and too little on the physical side. There we touch one of the immediate problems of education. We are still trying to strike a just balance between the two, and few educational experts would say that we have succeeded. Up to the present the mental curriculum has been seriously overloaded, and not very imaginatively designed in the classification of subjects; and while it is unjust to say, as Mr. Cahill alleges, that the Board of Education now pay very small attention to physical training, it is perfectly true that only recently have they and the subordinate education authorities become fully alive to the intimate and definite relation of "the broad back and the healthy body" to a normal healthy all-round education.

Some very wise and valuable things have been said by Sir George Newman, in recent publications by the Board, in reference to the provision of physical training, adapted to meet different ages and different types of school, and we have little doubt that before long a co-ordinate system will be developed which will begin in the infant schools and be carried right through to the adolescent stage in the new continuation schools. After listening to Mr. Cahill's strictures and theories, the Conference carried a resolution "viewing with the greatest alarm the postponement of the appointed date for the introduction of compulsory physical training under the Education Act, 1918." We share that alarm, and we are fully convinced that the postponement is in no way due to any desire on the part of the Board of Education to curtail the great schemes of the 1918 Act. Following the general demand for national economy, they are compelled for the time being to share the fate of the spendthrift departments of State, on which the pruning knife is said to be descending with ruthless severity.

